# A computational model of internal representations of chemical gradients in environments for chemotaxis of *Caenorhabditis elegans*

**DOI:** 10.1038/s41598-018-35157-1

**Published:** 2018-11-21

**Authors:** Zu Soh, Kazuma Sakamoto, Michiyo Suzuki, Yuichi Iino, Toshio Tsuji

**Affiliations:** 1 Department of System Cybernetics, Institute of Engineering, Hiroshima University, Higashi-Hiroshima, Hiroshima Japan; 2 Department of System Cybernetics, Graduate School of Engineering, Hiroshima University, Higashi-Hiroshima, Hiroshima Japan; 3 Sony Corporation, Minato-ku, Tokyo Japan; 4 Department of Radiation-Applied Biology Research, Takasaki Advanced Radiation Research Institute, National Institutes for Quantum and Radiological Science and Technology, Takasaki, Gunma Japan; 5 Department of Biological Sciences, Graduate School of Science, The University of Tokyo, Bunkyo-ku, Tokyo Japan

## Abstract

The small roundworm *Caenorhabditis elegans* employs two strategies, termed pirouette and weathervane, which are closely related to the internal representation of chemical gradients parallel and perpendicular to the travelling direction, respectively, to perform chemotaxis. These gradients must be calculated from the chemical information obtained at a single point, because the sensory neurons are located close to each other at the nose tip. To formulate the relationship between this sensory input and internal representations of the chemical gradient, this study proposes a simple computational model derived from the directional decomposition of the chemical concentration at the nose tip that can generate internal representations of the chemical gradient. The ability of the computational model was verified by using a chemotaxis simulator that can simulate the body motions of pirouette and weathervane, which confirmed that the computational model enables the conversion of the sensory input and head-bending angles into both types of gradients with high correlations of approximately *r* > 0.90 (*p* < 0.01) with the true gradients. In addition, the chemotaxis index of the model was 0.64, which is slightly higher than that in the actual animal (0.57). In addition, simulation using a connectome-based neural network model confirmed that the proposed computational model is implementable in the actual network structure.

## Introduction

The small roundworm *Caenorhabditis elegans* (*C. elegans*) represents one of the simplest model animals and is widely used to investigate neural computation. Its body consists of approximately 1000 cells, including 302 neurons with a known connectome^1^, as well as various types of sensory neurons and motor neurons whose anatomical structures were revealed in the 1970 s^2,3^, which allow the animal to sense external environments and select appropriate behavioural strategies for survival. Chemotaxis is one of the fundamental abilities of sensing chemical information and approaching a favourable environment based on the sensory information. *C. elegans* employs two types of behavioural strategies to perform chemotaxis. Pirouette is a strategy characterised by a series of behaviours starting with backward movement followed by sharp turns to change the travelling direction, and its occurrence depends on the chemical gradient parallel to the travelling direction of the body^4^. Weathervane represents a strategy of making a gradual curve toward a chemical peak, where the curving rate depends on the chemical gradient perpendicular to the travelling direction of the body^5^. If multiple sensory neurons are located spatially apart from each other, the gradients could be internally represented by the difference between their responses. However, the sensory neurons, for example, ASEL and ASER in the case of salt chemotaxis, are located in close proximity at the nose tip. Therefore, the neural network is required to calculate and internally represent two types of gradients using the temporal responses of the sensory neurons.

The input to a pair of interneurons (AIYL and AIYR) may play this role, as suggested by a study by Kocabas *et al*.^6^, which found that the symmetric input to the neurons changes the frequency of pirouette and that the asymmetric input controls the curving rate. In addition, model studies have predicted neural processing related to the weathervane strategy. Ferrée *et al*.^7^ constructed a simple nonlinear neural-network model to enable weathervane, and also extracted its computational rules using the impulse response of a linear neural network^8^. Morse *et al*.^9^ then verified the neural network model by navigating a robot to a light source. Izquierdo and Lockery^10^ subsequently proposed a simplified network structure that could adjust the curving rate from sensory inputs, and found that the nonlinearity and self-feedback of motor neurons may serve as key mechanisms for this function. Furthermore, their group demonstrated that the neural network model derived from the connectome was also able to perform weathervane^11^. Xu *et al*.^12^ proposed a dynamic neural-network model and simulated attraction and avoidance behaviour; in addition, their group recently combined a body dynamics model and a neural network model to enable chemotaxis^13^.

An understanding of chemotaxis acquired from the previous studies can be interpreted from the viewpoint of Marr’s level of analysis^14^. The behavioural analysis^4,5^ highlighted the problem at the computational level that the chemotaxis is closely related to the chemical gradients in the environments. The neural activity measurements^6^ revealed the phenomena at the implementational level. The simulation approaches^7–12^ analysed the information-processing mechanism at the algorithmic and implementational levels. However, these approaches did not directly treat the algorithm to calculate chemical gradients in the environments, which was explicitly or implicitly assumed given in the computational level. In other words, the relationships between the sensory input at the nose tip of *C. elegans* and internal representations of the two types of chemical gradients in the environments closely related to the observed strategies have not been formulated explicitly. Therefore, a gap exists between computational and implementational levels. Further, most previous models focused on revealing the mechanism of the weathervane and did not simulate the pirouette simultaneously.

This paper presents a simple and comprehensive computational model based on the motion of the animal involved in chemotaxis to bridge the gap of understanding between computational and implementational levels. The ability of the computational model to convert sensory inputs at the nose tip of *C. elegans* into internal representations of the chemical gradient parallel and perpendicular to the travelling direction was verified using a chemotaxis simulator that can simulate the body motions of pirouette and weathervane. The chemotaxis performance of the model was compared with previous experimental data^5^. For additional analysis of the implementational level, a connectome-based neural network model was constructed to test whether the computation could be implemented. Based on these results, we also discuss the relationship between the proposed computational model and the findings of the experimental^6^ and model^11^ studies.

## Results

### Chemical gradient with respect to the travelling direction derived from the NaCl concentration at the nose tip

Behavioural experiments revealed that pirouette and weathervane strategies are closely related to the chemical gradients parallel and perpendicular to the travelling direction^4,5^, respectively, but how can these gradients related to the traveling direction be obtained by using only the information accessible by the animal? To answer this question, we focused on the fact that ASEL/R responds to the time derivative of NaCl concentration, and the time derivative can be approximated by a directional derivative (see S2 Appendix 2 for the detailed derivation process). The internal representation of the gradients can then be obtained by decomposing the directional derivative of NaCl concentration sensed at the nose tip into the directional components parallel and perpendicular to the travelling direction. Based on this idea, a computational model is derived by using the directional derivative and first mean value theorem for definite integrals. The derived computational model is expressed by equations (1) and (2), to describe the relationships between the chemical concentration sensed at the nose tip and the internal representations of the chemical gradient in the environments, respectively.

1 $$\frac{d{y}_{p}}{dt}=-\,{a}_{p}{y}_{p}+{b}_{p}\frac{dc({{\boldsymbol{x}}}_{0},t)}{dt}+{{\epsilon }}_{p}$$

2 $$\frac{d{y}_{w}}{dt}=\{\begin{array}{c}-{a}_{w}{y}_{w}+{b}_{w}\frac{dc({{\boldsymbol{x}}}_{0},t)}{dt}+{{\epsilon }}_{w}\,\,({q}_{0} > 0)\\ -{a}_{w}{y}_{w}-{b}_{w}\frac{dc({{\boldsymbol{x}}}_{0},t)}{dt}+{{\epsilon }}_{w}\,\,({q}_{0}\le 0)\end{array}$$

where *y*_*p*_ and *y*_*w*_ are the internal representations of the gradients parallel and perpendicular to the travelling direction at the body centre, respectively, and *q*_0_ is the head-bending angle (cf. Materials and Methods; Multibody model); *a*_*p*_ and *a*_*w*_ are the reciprocals of the time constants that smooth the time derivative of the NaCl concentration sensed at the nose tip, and *b*_*p*_ and *b*_*w*_ are the gain constants to scale the inputs. The parameters in the model were adjusted and then set as follows: *a*_*p*_ = 0.58, *a*_*w*_ = 0.73, *b*_*p*_ = 1.20, *b*_*w*_ = 1.46. Because the derivation process involves several assumptions and approximations, we use ${{\epsilon }}_{p}$ and ${{\epsilon }}_{w}$ to express the accumulated approximation errors (see S2 Appendix 2).

The computational model can be interpreted as follows: Equation (1) works as a low-pass filter to eliminate the head-bending component, and equation (2) allows the comparison of the NaCl concentration between the ventral and dorsal sides using head-bending angles required to calculate the internal representation of the gradient perpendicular to the travelling direction (note that *C. elegans* lies on its side and navigates by dorsoventral motions).

To test the computational model, a chemotaxis simulation involving both weathervane and pirouette strategies was performed by using a multibody model of *C. elegans* and the chemical environmental model.

### Chemotaxis simulation using the multibody model

**Figure 1:**
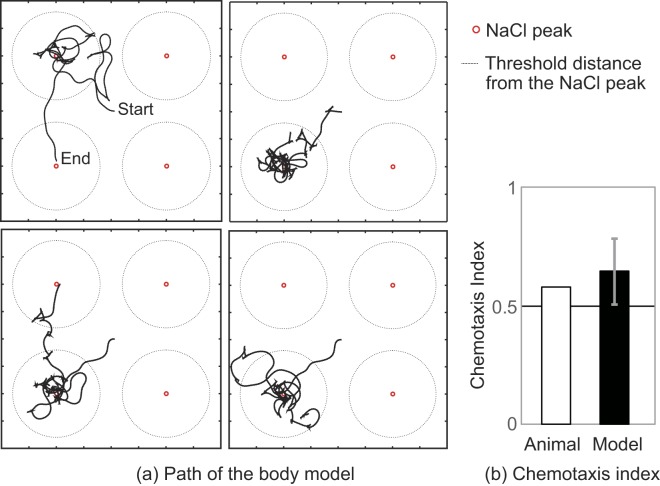
Chemotaxis simulation using the multibody model. (**a**) Schematic of four examples of chemotaxis paths out of 10 simulations. The body model was placed at the centre of the field and chemotaxis simulation was performed for 1200 s. The resultant paths of the body centre are shown as solid dark lines. The small red circles with the solid line indicate the NaCl peaks and the circles with the broken line indicate the threshold distance used to calculate the chemotaxis index^5^. (**b**) Chemotaxis indices of the simulation and the animal. The error bar represents the standard deviation of 10 simulations.

To evaluate the computational model represented by equations (1) and (2), it is necessary to determine the true chemical concentration at the nose tip and responses of sensory neurons (ASEL and ASER) as well as internal representations of the gradient. However, it is difficult to retrieve this information using experimental approaches. Therefore, we constructed a chemotaxis simulator including a multibody model that can perform motions related to pirouette and weathervane and an environmental model that can simulate the NaCl distribution on the agar plate (cf. Materials and methods, Chemotaxis simulator). This method enables the verification of the computational model at the behavioural level without measurement of internal representations of the gradients in the actual neural network. Figure 1a shows travelling path examples of the body centre obtained by chemotaxis simulation using the chemotaxis simulator. The chemotaxis simulation was repeated 10 times. In this simulation, the internal representation of the gradients parallel and perpendicular to the travelling direction were calculated using the computational model (equations (1) and (2)), and the calculated values were used to control the motion of the body model to perform pirouette and weathervane. The average travelling speed of the model was 1.28 ± 0.09 mm/s, which is consistent with the experimental data^5^. Figure 1b compares the chemotaxis index between the body model and the animals. Based on the previously defined chemotaxis index^5^, it is calculated as (*T*_in_ − *T*_out_)/*T*_total_, where *T*_in_ is the time spent within $\sqrt{(2/{\rm{\pi }})}$ cm from any NaCl peak, *T*_out_ is time spent outside the area, and *T*_total_ is the total simulation and experiment time, which is 1200 s in this case. The figure confirms a chemotaxis index of 0.64 ± 1.38 for the simulation. A comparison between the multibody model and the animal is presented in Supplemental Information S3 from the following aspects: the pirouette motion of the multibody model, a relationship between the curving rate and the gradient perpendicular to the traveling direction, a relationship between the bearing angles and the sharp turn angle, and evaluation of the error caused by converting body postures into the travelling path by using the multibody model.

**Figure 2:**
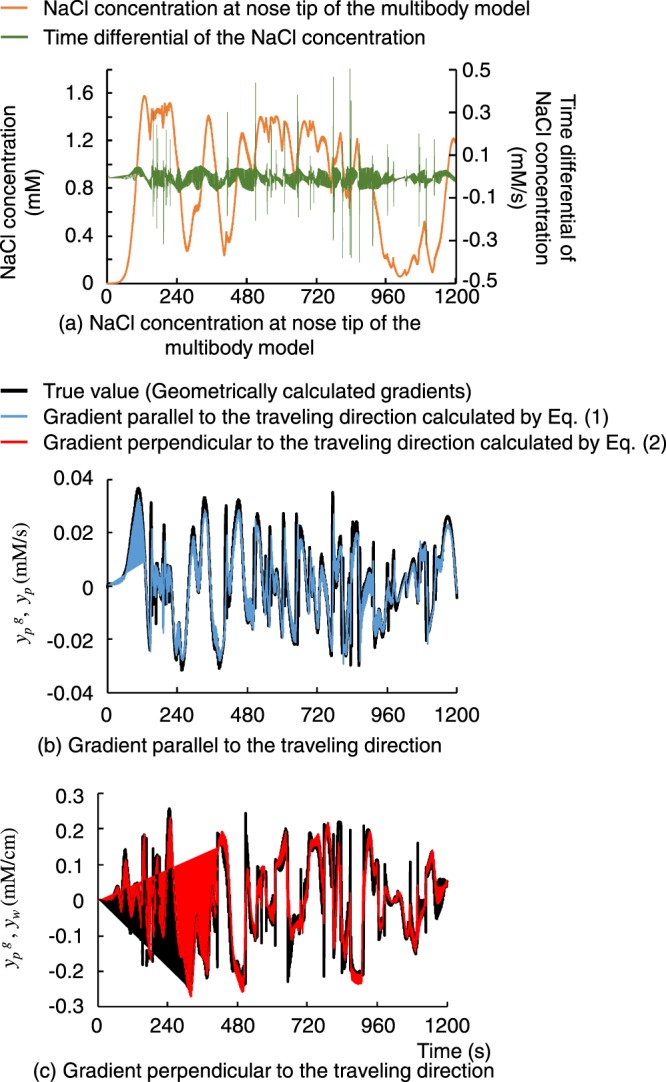
Comparison of the internal representation of the gradients generated by the computational model and geometrically calculated gradient (true value). Black lines indicate the true gradients geometrically calculated from the chemotaxis path shown in the upper left side of Fig. 1. (**a**) NaCl concentrations at the nose tip of the multibody model and time differential of the concentration corresponding to *dc*/*dt* in equations (1) and (2). Spikes observed in the time differential of the NaCl concentration are caused by backward movement for the initiation of pirouette. (**b**) Comparison of the gradient parallel to the travelling direction. The correlation is *r* = 0.91 (*p* < 0.01) and RMSE is 1.78 × 10^−6^ mM/s. (**c**) Comparison of the gradient perpendicular to the travelling direction. The correlation is *r* = 0.89 (*p* < 0.01) and RMSE is 0.15 × 10^−3^ mM/cm.

The true values of the gradients parallel and perpendicular to the travelling direction, denoted as ${y}_{p}^{G}$ and ${y}_{w}^{G}$, respectively, were then geometrically calculated based on the chemotaxis simulation path of the body centre, using equations (14)–(17), as described in Materials and Methods (Geometrical calculations of NaCl gradient), and compared with the internal representation of the gradients calculated using the computational model (equations (1) and (2)). Figure 2 shows comparison examples. The figure confirms a high correlation of *r* = 0.91 (*p* < 0.01) and *r* = 0.89 (*p* < 0.01) for the gradients parallel and perpendicular to the travelling direction, respectively. The average correlations over 10 chemotaxis simulations were *r* = 0.90 ± 0.03 and *r* = 0.91 ± 0.02, and the average root mean square error (RMSE) values were 1.71 ± 0.32 × 10^−6^ mM/s and 0.14 ± 0.01 × 10^−3^ mM/cm, for the respective gradients.

**Figure 3:**
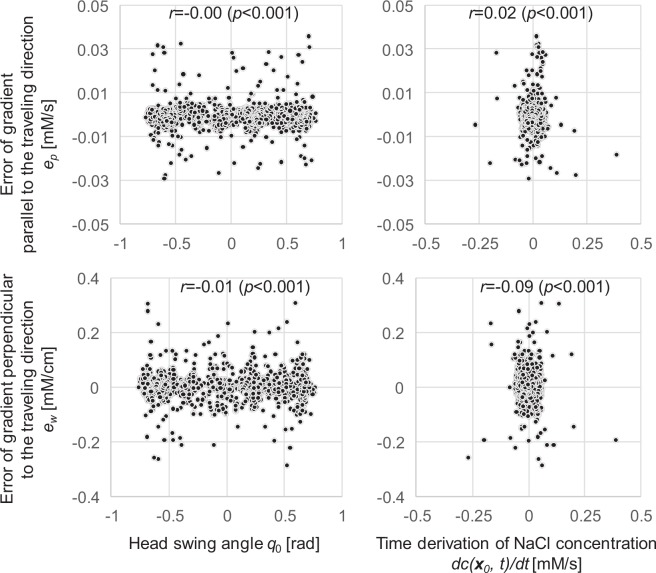
Input dependency of error in gradients parallel and perpendicular to the traveling direction. Errors between the gradients calculated geometrically and those generated by the computational model are plotted against the inputs to the computational model. The errors and inputs are obtained from the chemotaxis simulation results shown in the upper left part of Fig. 1a.

The computational model was derived based on several assumptions and approximations regarding the inputs. We thus tested input (*q*_0_, *dc*(***x***_0_, *t*)/*dt*) dependency of the errors, as shown in Fig. 3. Here, the errors are defined as ${e}_{p}={y}_{p}^{G}-{y}_{p}$ and ${e}_{w}={y}_{w}^{G}-{y}_{w}$, respectively, for the gradient parallel and perpendicular to the travelling direction. The figure confirms low correlations between the inputs and the errors. The average correlations of the 10 simulation results are as follows:

- *q*_0_ vs *e*_*p*_: 0.01 ± 0.02
- *dc*(***x***_0_, *t*)/*dt* vs *e*_*p*_: 0.08 ± 0.05
- *q*_0_ vs *e*_*w*_: 0.01 ± 0.02
- *dc*(***x***_0_, *t*)/*dt* vs *e*_*w*_: 0.1 ± 0.06

*p < 0.001 for all correlations.

These results demonstrate low correlations between the errors and inputs, indicating almost no input dependency.

### Generating internal representations of the chemical gradient based on the neural network structure of *C. elegans*

**Figure 4:**
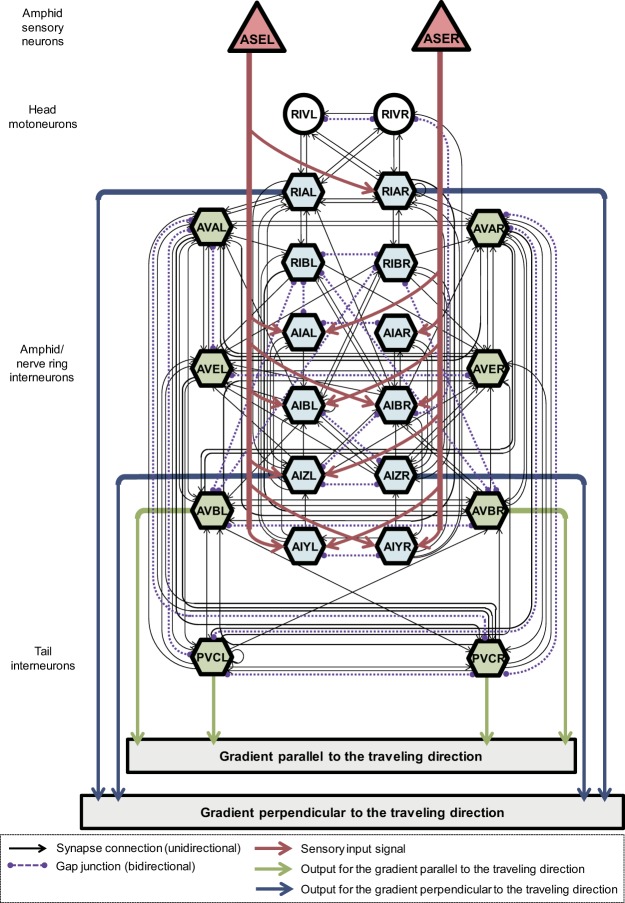
Structure of the neural network model regarding to chemotaxis in *C. elegans*.

The computational model indicates that generating the internal representation of NaCl gradients requires the time derivative of NaCl concentration at the nose tip and head-bending angle. We thus tested whether the connectome-based neural network exhibited the ability to generate the internal representations from this information. A neural network model was constructed based on the neural connection structure derived from WormAtlas^15^ and neurons included in the model are were chosen based on those treated in Iino and Yoshida^5^. The simulation results were then compared with the computational model described by equations (1) and (2). The structure of the network model is shown in Fig. 4.

The model considers the response characteristics of sensory neurons ASEL and ASER using equations (20)–(21) (cf. Materials and Methods, Neural network model) based on measured data^16^. In addition, although previous studies^4,6,7^ suggested that the gradients are not directly coded in the neural network of *C. elegans*, to facilitate the evaluation of the implementability of the computational model in the neural network, we assumed that the interneurons explicitly represent these gradients. In this case, PVC(L/R) and AVB(L/R) interneurons were assumed to output the gradient parallel to the travelling direction because these neurons are responsible for controlling forward motion^17^ and may inhibit backward motion followed by a sharp turn. In addition, the RIA(L/R) and AIZ(L/R) interneurons were assumed to output the gradient parallel to the travelling direction because these neurons control head bending^1,18^, which can generate turning bias. The model considers both chemical synapse connections and gap junctions; these connection weight parameters were trained using back-propagation through a time algorithm^19^ modified to simultaneously train the parameters of both gap junctions and chemical synapses. The training datasets were generated using data from the chemotaxis simulation performed using the multibody model, wherein the inputs were the NaCl concentration sensed at the nose tip of the multibody model and the head-bending angles, and the teacher signals comprised true chemical gradients that were geometrically calculated from the path of the body centre obtained from the chemotaxis simulation.

**Figure 5:**
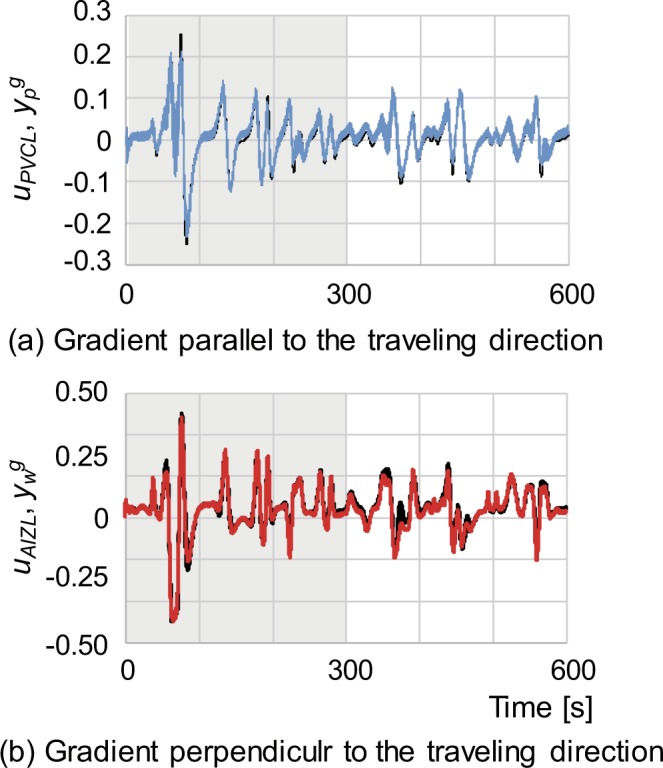
Comparison between the outputs of the connectome-based neural network (coloured lines) and true values of gradients (black lines). The y-axis shows the NaCl gradient normalised to a range of [−0.5, 0.5] and the x-axis represents time in seconds. The grey highlights indicate the time interval (from 0 to 300 s) used as training data, and the remaining intervals are for validation. (**a**) Comparison of the output of PVCL and the gradient parallel to the travelling direction. (**b**) Comparison of the output of AIZL and the gradient perpendicular to the travelling direction.

Figure 5a,b show an example of comparison results between the output of the trained neural network model and the true chemical gradients, where the data in the time interval of 0–300 s are used for training the neural network and the data in the remaining 300 s are used to validate generality. The result confirmed the correlations of *r* = 0.94 (*p* < 0.01) and *r* = 0.99 (*p* < 0.01) for the gradients parallel and perpendicular to the travelling direction in the training interval, and *r* = 0.92 (*p* < 0.01) and *r* = 0.95 (*p* < 0.01) for the gradients parallel and perpendicular to the travelling direction in the validation interval, respectively. The average correlations over 10 chemotaxis simulation datasets were *r* = 0.91 ± 0.04 and *r* = 0.97 ± 0.03 for the gradients parallel and perpendicular to the travelling direction in the training interval, and *r* = 0.89 ± 0.04 and *r* = 0.91 ± 0.03 for the gradients parallel and perpendicular to the travelling direction in the validation interval, respectively. It should be noted that the gradients were normalised to a range of [−0.5, 0.5] considering the output range of the sigmoid function used in the neural network model.

**Figure 6:**
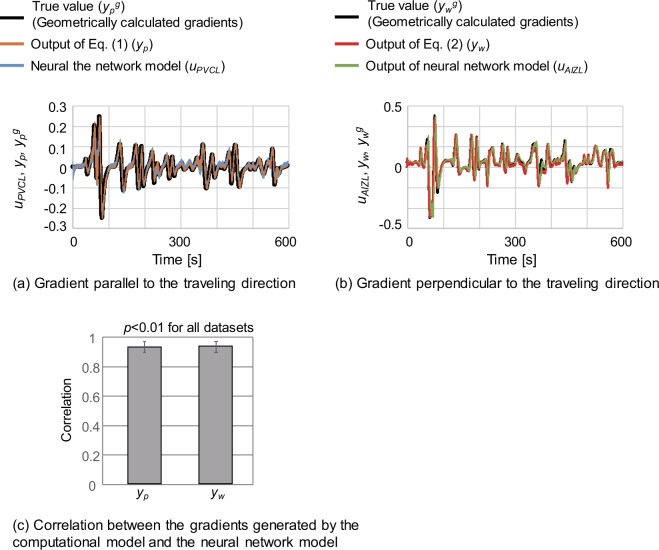
Comparison among the true gradients, the outputs of the neural network model, and the outputs of the computational model. (**a**) Shows the comparison of the gradients parallel to the travelling direction. (**b**) Shows the comparison of the gradients perpendicular to the travelling direction. (**c**) Shows the average correlation between the neural network model and the computational model. The average correlations were calculated using 10 chemotaxis simulation results partially shown in Fig. 1. The error bars represent the standard deviations.

Finally, we compared the computational model with the neural network model. For this comparison, the outputs of the computational model (equations (1) and (2)) were calculated for the 10 chemotaxis-simulation datasets using the previously described parameters. Figure 6a,b show examples of the outputs of the neural-network model and the computational model. Figure 6c shows the correlations between the outputs of the neural network model and those of the computational model where the average correlations were *r* = 0.93 ± 0.04 for both gradients parallel and perpendicular to the travelling direction and *p* < 0.01 for all simulation datasets.

## Discussion

### Relationship between NaCl concentration at the nose tip and the internal representations of chemical gradients

This paper presents the computational model that can describe the relationships between the chemical concentration sensed at the nose tip of *C. elegans* and the internal representations of chemical gradients closely related to pirouette and weathervane strategies, and tests if the computational model is implementable in the connectome-based neural network model. This analysis concept is based on Marr’s level of analysis where the computational model corresponds to the analysis on the computational level, and the neural network simulation corresponds to the analysis on the implementational level.

To verify the computational model, we constructed a chemotaxis simulator which includes the environment model expressing NaCl distribution and the multibody model of *C. elegans*. The results shown in Fig. 1 and the supplemental data indicate that the chemotaxis simulator has the ability to simulate the behaviour during chemotaxis, and is therefore applicable for testing equations (1) and (2). Figure 2 then confirms that equations (1) and (2) could convert the NaCl concentration sensed at the nose tip into the internal representation of the NaCl gradient parallel and perpendicular to the travelling direction. Equation (2) indicates that generating the internal representation of the gradient perpendicular to the travelling direction requires the head-bending angle. It is commonly considered that head bending is generated by a central pattern generator^20^ involving stretch receptors^21^; thus, its information may be obtained through these neurons.

The derivation process (see S2 Appendix 2) shows that the directional decomposition of the time derivative of NaCl concentration requires cosine (symmetric) and sine (asymmetric) functions of the head-bending angle, respectively, for parallel and perpendicular to the travelling direction. Similar decomposition process could be performed in the pair of AIY neurons or their upstream neurons because the results of an experimental study^6^ indicated that the symmetric input of the pair of AIY neurons controls the pirouette frequency and the asymmetric input controls the gradual turn (weathervane).

In addition, one of the findings of the model study by Izquierdo & Beer^11^ indicated that the asymmetrical response characteristics of the ventral and dorsal motor neurons are required to perform gradual turns (weathervane). That is, the response characteristic of either side of the motor neuron would be shifted to the region of lower sensitivity and the other side to that of higher sensitivity, by the sensory input, so that the motor neurons generate a biased sinusoidal wave to regulate body motion. From the computational model (equation (2)), it can be interpreted that the motor neurons perform the directional decomposition, and the gradient parallel to the travelling direction is coded in the motion of the animal.

As described above, the proposed computational model suggests that both findings obtained from the observation of AIY neurons and motor neurons in the simulation could be explained by directional decomposition. From the viewpoint of Marr’s level of analysis, the computational model can bridge the gap between the problem defined in the computational level and implementational level.

### Generating internal representations of chemical gradients based on the neural network structure of *C. elegans*

Figure 5 confirms that the training algorithm successfully adjusted the parameters of the neural network model and that it could internally represent gradients with respect to the travelling direction using the NaCl concentration sensed at the nose tip and the head-bending angle. High correlations between the respective gradients generated by the computational model, neural network model, and geometrical calculation (shown in Fig. 6) indicate that the computational model can be implemented in the connectome-based neural network.

### Limitation

In the neural network model, AVB and PVC were assumed to output the gradient parallel to the travelling direction. However, in the measurement experiments, activities in these neurons were observed despite the absence of a gradient^22^, which indicates that AVB and PVC are redundant in the calculation of the gradients. We confirmed this redundancy by excluding AVB and PVC from the connectome-based neural network model, setting AIY as the output neuron, and readjusting the parameters. Accordingly, AIY generated the gradient parallel to the travelling direction with a correlation of 0.98 (*p* < 0.001), indicating that the computational model could be implemented with various parameter sets. This redundancy in the parameter space limits the analysis of the contribution of the respective neuron to chemotaxis.

The parameters of the multibody model and motions such as body forms and reversal durations are chosen such that the body model can approximate the motions of the actual animal as close as possible (see Supplemental Information S3). Although the approximation error affects the analysis results, the error of generated gradients (see Fig. 2) caused by the multibody model may be partial (see Fig. S5 in Supplemental Information S3). The modelling error, however, is inevitable in a process to approximate the motion of *C. elegans*. To reduce the modelling error, merging the simulation and experimental approaches can be a candidate solution for future research. In addition, the motion of the multibody model was given by a sinusoidal function, as shown in equations (5) and (6) in Material and Methods: Chemotaxis simulator. Thus, the model ignores small random wriggling motions of the nose tip that can be observed in the actual animal. As the sensory neurons are located at the nose tip, this motion can affect the sensory inputs. However, this evaluation is not performed in this study.

## Conclusion

In this study, a simple and comprehensive computational model was derived to convert the response of a single sensory input into two types of internal representations of the NaCl gradient parallel and perpendicular to the travelling direction and enabled simultaneous simulation of the pirouette and weathervane strategies. The derived computational model suggests that internal representations of the gradients can be generated by combining head-bending angles and sensory input from ASEL/R neurons. It could also be used to interpret the functions of AIY neurons and motor neurons, respectively, identified in previous experimental^6^ and simulation studies^11^, and thus can bridge the gap between the chemotaxis problems at the computational and implementational levels.

The connectome-based neural network model included in the chemotaxis simulator demonstrated that the computational model could be implemented in it, although the coding manner of the chemical gradient might differ from that of the actual animals. The connectome-based neural network model may allow further analysis of the functions of respective neurons by introducing the biological constrictions and measured neural activities and by simulating ablation experiments.

## Materials and Methods

To evaluate the derived computational model [equations (1) and (2)], we developed a chemotaxis simulator including the multibody and environmental models. The body model can perform both weathervane and pirouette motion. The NaCl chemical gradients parallel and perpendicular to the travelling direction in an environment, which are called a true gradient in this paper, were calculated from the simulated body centre path. To test the implementability of the proposed computational model under actual neural structure representations, a connectome-based neural network model was constructed. All simulations described in this study were performed using MATLAB 2013. Model parameters are given in S1 Appendix 1. The following text explains each part of the chemotaxis simulator, simulation method, and neural network model.

### Chemotaxis simulator: Multibody model

**Figure 7:**
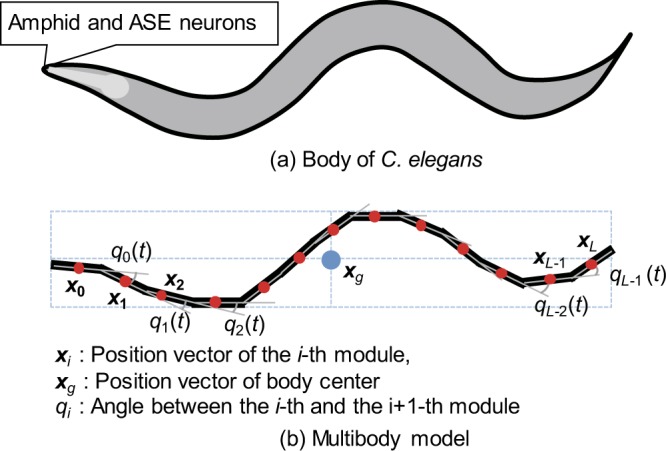
Body of *C. elegans* and the multibody model.

*C. elegans* body was approximated using a multibody model, as shown in Fig. 7, defined by the following Newton-Euler equations based on a previous study^23^:

3 $${\boldsymbol{I}}({\boldsymbol{q}})\frac{{d}^{2}{\boldsymbol{x}}}{d{t}^{2}}+{\boldsymbol{h}}({\boldsymbol{q}},\frac{d{\boldsymbol{q}}}{dt})+{\boldsymbol{g}}({\boldsymbol{q}})={\boldsymbol{\rho }}+\sum _{j}\,{{\boldsymbol{J}}}_{{\boldsymbol{j}}}^{{\bf{T}}}{{\boldsymbol{F}}}_{{\boldsymbol{j}}},$$

4 $${{\boldsymbol{I}}}_{{\rm{g}}}({\boldsymbol{q}})\frac{{d}^{2}{{\boldsymbol{x}}}_{{\boldsymbol{g}}}}{d{t}^{2}}+{{\boldsymbol{h}}}_{{\rm{g}}}({\boldsymbol{q}},\frac{d{\boldsymbol{q}}}{dt})+{{\boldsymbol{g}}}_{{\rm{g}}}({\boldsymbol{q}})=\sum _{j}\,{{\boldsymbol{F}}}_{{\boldsymbol{j}}},$$

where ***x*** = [*x*_*i*_, *y*_*i*_]^**T**^ represents the *i*-th body centroid position, ***x***_***g***_ = [*x*_*g*_, *y*_*g*_]^**T**^ represents the body centre position, ***q*** = [*q*_0_, *q*_1_, …, *q*_*L*−1_]^T^ represents the angles between adjacent modules, ***I***(***q***) and ***I***_g_(***q***) represent the inertia matrix, ${\boldsymbol{h}}({\boldsymbol{q}},\frac{d{\boldsymbol{q}}}{dt})$ and ${{\boldsymbol{h}}}_{{\rm{g}}}({\boldsymbol{q}},\frac{d{\boldsymbol{q}}}{dt})$ are centrifugal and Coriolis force terms, ***g***(***q***) and ***g***_***g***_(***q***) are gravity force terms, ***ρ*** = [*ρ*_0_, *ρ*_1_, …, *ρ*_*L*−1_]^**T**^ is the driving torque generated from the motors, ***F***_***j***_ = [*F*_*T*,*j*_, *F*_*N*,*j*_] is the friction vector between the floor and the *j*-th body, and ***J***_***j***_ is the Jacobian matrix. Please note that we omit the explicit notation of time dependence of the variables for simplification, but all variables depend on time in equations (3) and (4). The parameters related to inertia, centrifugal, Coriolis, and gravity forces are determined based on animal size and weight (Tables in S1 Appendix 1). The friction forces were determined based on the average velocity of the animal on the chemotaxis plate (0.12 mm/s)^5^.

### Chemotaxis simulator: implementing pirouette and weathervane

Body model motion is controlled by a sinusoidal function determining each *i*-th joint angle *q*_i_(*t*).

5 $${q}_{i}(t)={q}_{{\rm{Max}}}\,\sin \,(\omega t-{\varphi }_{i}(t))+\kappa (t),$$

6 $${\varphi }_{i}(t)=-\,2\pi \psi \,(i-1),$$

where *q*_Max_ is the maximum joint bending angle, *ω* determines the angular velocity of bending, *ϕ*_*i*_(*t*) determines the wavelength of the animal body as well as switching between forward and backward motion, and *ψ* (in rad) is determined by the measured body wavelength^24^. The bias parameter *κ*(*t*) = *κ*_*w*_(*t*) + *κ*_*R*_(*t*) is determined by the weathervane turning rate *κ*_*w*_(*t*) and random walk *κ*_*R*_(*t*). Pirouette and weathervane were implemented by changing *ϕ*_*i*_(*t*) and *κ*(*t*) as follows:

*Weathervane*.

7 $${\kappa }_{w}\,(t)=-\,{C}_{w}{d}_{s}\,(t)$$

where *d*_*s*_(*t*) (in mM/mm) represents the gradient perpendicular to the traveling direction and *C*_*w*_ is derived from the measured weathervane curvature^5^.

#### Pirouette

Pirouette frequency *f*_*p*_(*t*) (in events/s) is given by the following equation^5^:

8 $${f}_{p}\,(t)=\frac{0.023}{0.4+\exp \,(140{d}_{t}(t))}+0.0033$$

where *d*_*t*_(*t*) (in mM/s) is the gradient parallel to the travelling direction. A uniform random number *R*(*t*) ∈ [0, 1] is generated every sampling time and pirouette is initiated if *R*(*t*) < *f*_*p*_(*t*).

The pirouette starts with backward motion by switching the phase *ϕ*_*i*_(*t*) using the following equation:

9 $${\varphi }_{i}(t)=\pi +2\omega {T}_{p0}-{\varphi }_{i}({T}_{p0})$$

where *T*_*p*0_ is the pirouette initiation time. The backward motion lasts for *T*_*b*_ = 6.0 s because measurement data indicate that most sharp turns occur after a long reversal with around three head-bending cycles^25^ and their probability is approximately 90% after 6.0 s of backward motion^26^. When the backward motion is finished, equation (9) is applied again to switch back to forward motion.

A sharp turn is then initiated after the backward motion using the following equation:

10 $${\varphi }_{i}\,(t)=\{\begin{array}{l}{\varphi }_{i}\,(t-{\rm{\Delta }}t)+\frac{{C}_{p}}{{T}_{P1}}{\rm{\Delta }}t,\,{T}_{{\rm{\Omega }}0} < t < {T}_{{\rm{\Omega }}1}\,\\ \,{\varphi }_{i}\,(t),\,\,\,{T}_{{\rm{\Omega }}1} < t < {T}_{{\rm{\Omega }}2}\\ {\varphi }_{i}\,(t-{\rm{\Delta }}t)-\frac{{C}_{p}}{{T}_{P1}}{\rm{\Delta }}t,\,{T}_{{\rm{\Omega }}2} < t < {T}_{{\rm{\Omega }}3}\end{array}$$

where *T*_Ω0_ = *T*_*p*0_ + *T*_*b*_ is the sharp turn initiation time, *T*_Ω1_ = *T*_Ω0_ + *T*_*p*1_, *T*_Ω2_ = *T*_Ω1_ + *T*_*p*2_, and *T*_Ω*E*_ = *T*_Ω2_ + *T*_*p*3_ are duration parameters for linearly changing the *ϕ*_*i*_(*t*), and *C*_*p*_ is the maximum phase delay. The total sharp turn duration is set to *T*_Ω*E*_ − *T*_Ω0_ = 3.18 s, and *C*_*p*_ was adjusted to generate turns larger than 1.75 rad based on definitions from a previous study^5^. Temporal changes in *φ*_*i*_(*t*) allow the body shape to change from the S shape to the Ω shape and back, as illustrated in Fig. S2 in Supplemental Information S3.

#### Random walk

The random walk was introduced based on measured curvature in the absence of a chemical gradient as in the following equations:

11 $${\kappa }_{T}=N(0,{C}_{r})$$

12 $${\kappa }_{R}\,(t)={\kappa }_{R}\,(t-{\rm{\Delta }}t)+\frac{{\kappa }_{T}-{\kappa }_{T-1}}{{\rm{\Delta }}T}$$

Based on simulations performed by Iino and Yoshida^5^, a random curvature parameter *b*_*T*_ was chosen every Δ*T* = 12 s and linearly altered for every sampling time Δ*t* (in s).

#### Chemotaxis simulator: Environmental model

The environmental model and its parameters were derived from a previous study^5^. NaCl concentration at an arbitrary position ***x*** can be calculated by solving Fick’s equation as follows:

13 $$c\,(x,t)={N}_{0}\sum _{k=1}^{K}\,\frac{\exp (-\frac{|{\boldsymbol{x}}-{{\boldsymbol{x}}}_{k}|}{{r}_{1}Dt})}{{r}_{2}EDt}$$

where *N*_0_ is the NaCl solution concentration, *D* is the NaCl diffusion coefficient, *E* is the agar plate thickness, and ***x***_*k*_ is the coordinate of the *k*-th NaCl point.

Chemotaxis simulations were performed after 3600 s of NaCl diffusion, with each being performed for 1200 s and repeated 10 times using the multibody model. Notably, NaCl continued to diffuse during the simulation based on equation (13).

### Chemotaxis simulator: Simulation procedure and gradient calculation

#### Simulation

Chemotaxis simulation was performed according to the following procedure, wherein the time step was set to 0.01 s.

1. The multibody model was placed in the environmental model centre [***x***_***g***_**=**(*x*_g_, *y*_g_)=(0, 0)].
2. The multibody model posture was calculated by using equations (5) and (6).
3. The dynamics were calculated by solving equations (3) and (4) and used to update the current position ***x***_***g***_.
4. NaCl concentration at the nose tip (***x***_***0***_) was calculated by using equation (13).
5. Nose tip NaCl concentration was converted into the internal representation of the gradients parallel and perpendicular to the travelling direction by using the proposed computational model (equations (1) and (2)).
6. Pirouette initiation was judged by using equation (8)
7. Pirouette was simulated by using equations (9) and (10); otherwise, weathervane was simulated by using equation (7) and random walk by using equations (11) and (12).
8. Steps 2–7 were iteratively implemented for each succeeding time step until the total simulation time of 1200 s was attained.

### Geometrical calculation of NaCl gradient

Using the environmental model, the true gradient parallel to the travelling direction ${y}_{p}^{g}$ was geometrically calculated as follows:

14 $${y}_{p}^{g}=\frac{c({{\boldsymbol{x}}}_{{\boldsymbol{g}}}(t),t)-c({{\boldsymbol{x}}}_{{\boldsymbol{g}}}(t-{\rm{\Delta }}t),t-{\rm{\Delta }}t)}{{\rm{\Delta }}t},$$

The true gradient to the travelling direction ${y}_{w}^{g}$ was also geometrically calculated as follows:

15 $${y}_{w}^{g}=\frac{c({{\boldsymbol{x}}}_{{\boldsymbol{g}}}(t)+{\rm{\Delta }}{{\boldsymbol{x}}}_{{\boldsymbol{v}}},t)-c({{\boldsymbol{x}}}_{{\boldsymbol{g}}}(t)+{\rm{\Delta }}{{\boldsymbol{x}}}_{{\boldsymbol{d}}},t)}{2\delta },$$

16 $${\rm{\Delta }}{{\boldsymbol{x}}}_{{\boldsymbol{v}}}(t)=\delta \frac{[\begin{array}{cc}0 & 1\\ -1 & 0\end{array}]({{\boldsymbol{x}}}_{{\boldsymbol{g}}}(t)-{{\boldsymbol{x}}}_{{\boldsymbol{g}}}(t-{\rm{\Delta }}t))}{|{{\boldsymbol{x}}}_{{\boldsymbol{g}}}(t)-{{\boldsymbol{x}}}_{{\boldsymbol{g}}}(t-{\rm{\Delta }}t)|},$$

17 $${\rm{\Delta }}{{\boldsymbol{x}}}_{{\boldsymbol{d}}}(t)=-\,\delta \frac{[\begin{array}{cc}0 & 1\\ -\,1 & 0\end{array}]({{\boldsymbol{x}}}_{{\boldsymbol{g}}}(t)-{{\boldsymbol{x}}}_{{\boldsymbol{g}}}(t-{\rm{\Delta }}t))}{|{{\boldsymbol{x}}}_{{\boldsymbol{g}}}(t)-{{\boldsymbol{x}}}_{{\boldsymbol{g}}}(t-{\rm{\Delta }}t)|},$$

where ***x***_***g***_(*t*) − ***x***_***g***_(*t* − Δ*t*) is the body centre travelling direction and *δ* is a small displacement; thus Δ***x***_***v***_(*t*) and Δ***x***_***d***_(*t*) are the vectors perpendicular to the travelling direction.

### Neural network model

The neural network model shown in Fig. 4 was defined based on the actual connection structure derived from WormAtlas^15^ considering both synaptic connections and gap junction, and neurons included in the model were derived from Iino and Yoshida^5^. The model receives inputs of the head-bending angles and NaCl concentration at the nose tip of the multibody model from RIV(L/R) and amphid sensory neurons ASE(L/R), respectively. The ASEL and ASER responses are determined from the previous experimental data^16^ and sent to the interneurons in both amphid and nerve ring. To facilitate the evaluation of implementability of the computational model on the neural network, we assumed that the interneurons could directly generate the gradients, although previous studies^6,10,11^ have suggested that these gradients are not directly coded in the *C. elegans* neural network. In this case, PVC(L/R) and AVB(L/R) interneurons were assumed to output the gradient parallel to the travelling direction because these neurons are responsible for controlling forward motion^17^ and may inhibit backward motion followed by a sharp turn. Additionally, interneurons RIA(L/R) and AIZ(L/R) were assumed to output the gradient perpendicular to the travelling direction because these neurons control head bending^1,13^, which can generate turning bias. The following equations give a mathematical definition of the neural network model:

The dynamic characteristic of a neuron is given as follows:

18 $$\begin{array}{rcl}{u}_{i}(t+1) & = & \tfrac{1}{1+{T}_{s}{\tau }_{i}}{u}_{i}(t)+\tfrac{{T}_{s}}{1+{T}_{s}{\tau }_{i}}\{\sum _{j}^{n+m}\,{w}_{ij}{z}_{j}(t)+\sum _{j}^{n}\,{s}_{ij}({u}_{j}(t)-{u}_{i}(t))\}\\  &  & +\,\tfrac{1}{1+{T}_{s}{\tau }_{i}}\{\sum _{j}^{n+m}\,{w}_{ij}^{d}({z}_{j}(t)-{z}_{j}(t-1))\},\end{array}$$

19 $${U}_{i}(t+1)=\frac{1}{1+\exp (-{u}_{i}(t+1))},$$

where *u*_*i*_(*t*) corresponds to the electrical current to the *i*-th neuron, *U*_*i*_(*t*) corresponds to the membrane potential, *T*_*s*_ is the sampling time, *τ*_*i*_ is the time constant caused by leakage current, *w*_*ij*_, and ${w}_{ij}^{d}$ are the chemical synapse connection strengths from the *j*-th to the *i*-th neuron, *s*_*ij*_ = *s*_*ji*_ is the conductance of a gap junction, *n* is the total number of neurons, and *m* is the total input number. Additionally, *z*_*j*_(*t*) = *u*_*j*_(*t*) when *j* ≤ *n*, and *z*_*j*_(*t*) = *I*_*j*−*n*_(*t*) when *n* < *j* ≤ *n* + *m* where *I*_*j*_(*t*) is the external input including NaCl concentration and head-bending angle *q*_0_(*t*).

**Figure 8:**
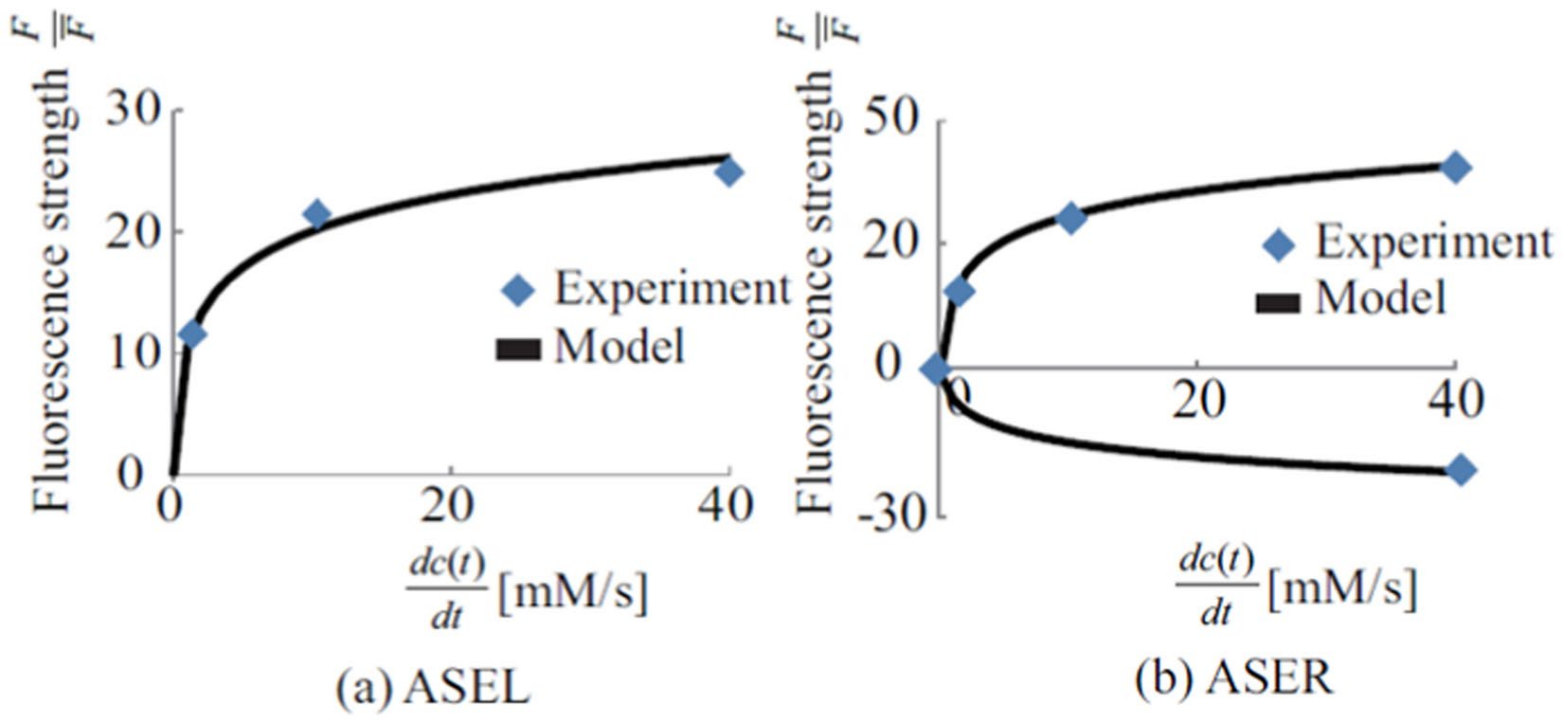
Measured peak responses and the input to the model. (**a**) Shows the input to ASEL. (**b**) Shows the input to ASER.

ASER and ASEL neurons were modelled based on the experimental data^16^ as follows:

20 $${I}_{1}(t)=\{\begin{array}{c}{a}_{L}\,\mathrm{log}({b}_{L}\frac{dc}{dt}+1),\,\frac{dc}{dt} > 0\\ 0\,\,\,\,\,\frac{dc}{dt}\le 0\end{array},$$

21 $${I}_{2}(t)=\{\begin{array}{ll}{a}_{R}^{+}\,\mathrm{log}({b}_{R}^{+}\frac{dc}{dt}+1),\, & \frac{dc}{dt} > 0\\ {a}_{R}^{-}\,\mathrm{log}({b}_{R}^{-}\frac{dc}{dt}+1) & \frac{dc}{dt}\le 0\end{array},$$

where *I*_1_(*t* + 1) and *I*_2_(*t* + 1) represent inputs to ASEL and ASER neurons, respectively. *dc*/*dt* is the NaCl concentration time derivative at the nose tip. The parameters ${a}_{L},\,{b}_{L},\,{a}_{R}^{+},\,{b}_{R}^{+},\,{a}_{R}^{-},\,{b}_{R}^{-}$ are adjusted to fit the response peaks of ASEL and ASER neurons. Figure 8 shows the fitting results, which confirms that the model could generate a response similar to the experimental data^16^.

Adjustments of chemical synapse connections *w*_*ij*_ and gap junctions *s*_*ij*_ were performed using back-propagation through a time algorithm, which uses the chain-rule of partial differential on the following evaluation function:

22 $$H=\frac{1}{2}\sum _{t=1}^{T}\,\sum _{i=1}^{n}\,{\mu }_{i}{({U}_{i}(t)-{d}_{i}(t))}^{2}$$

where *μ*_*i*_ = 1 if index *i* corresponds to the neuron that outputs a gradient either parallel or perpendicular to the traveling direction; otherwise *μ*_*i*_ = 0, *d*_*i*_(*t*) is the target gradient that is desired to be outputted from the corresponding neuron. The chemical gradient is normalised to produce *d*_*i*_(*t*) by using the following equations as the output values of a neuron model are in the range of [0, 1].

23 $${d}_{i}(t)=\frac{{c}_{\zeta }(t)-\mathop{{\rm{\min }}}\limits_{t}({c}_{\zeta }(t))}{\mathop{{\rm{\max }}}\limits_{t}({c}_{\zeta }(t))-\mathop{{\rm{\min }}}\limits_{t}({c}_{\zeta }(t))},$$

where *c*_*ζ*_ is a gradient either parallel or perpendicular to the traveling direction. The parameters *w*_*ij*_, *s*_*ij*_, *τ*_*i*_ are then iteratively updated based on the partial differential of *H* by the following equations:

24 $${w}_{ij}\leftarrow {w}_{ij}-{\eta }_{w}\frac{\partial H}{\partial {w}_{ij}},$$

25 $${s}_{ij}\leftarrow {s}_{ij}-{\eta }_{g}(\frac{\partial H}{\partial {s}_{ij}}+\frac{\partial H}{\partial {s}_{ji}}),$$

26 $${\tau }_{i}\leftarrow \{\begin{array}{cc}{\tau }_{i}-{\eta }_{\tau }\frac{\partial H}{\partial {\tau }_{i}}, & {\tau }_{i} > 0\\ 0 & {\tau }_{i}\le 0\end{array},$$

where *η*_*w*_, *η*_*g*_, *η*_*τ*_ represent the learning rate. As shown in the above equations, the gap junction is constrained to *s*_*ij*_ = *s*_*ji*_, and the time constant is constrained to *τ*_*i*_ > 0.

## Electronic supplementary material

Appendix
