# Supplementary material for: A computational model of internal representations of chemical gradients in environments for chemotaxis of Caenorhabditis elegans
Source: Sci Rep. 2018 Nov 21;8:17190. doi: 10.1038/s41598-018-35157-1 (PMC6249258; doi:10.1038/s41598-018-35157-1)
Supplement: Supplementary file 1 — Appendix [file 41598_2018_35157_MOESM1_ESM.docx]

**S1 Appendix 1**

**A computational model of internal representations of chemical gradients in environments for chemotaxis of *Caenorhabditis elegans***

Zu Soh, Kazuma Sakamoto, Michiyo Suzuki, Yuichi Iino, Toshio Tsuji

Table S1 lists the symbols used in Eqs. (1) and (2).

**Table S1. Symbols used in the proposed computational model**

| Symbol | Interpretation |
| --- | --- |
| $y_{p}$, $\dot{y}_{p}$ | Gradient parallel to the traveling direction and its time derivative |
| $a_{p}$, $b_{p}$ | Parameters to calculate the gradient parallel to the traveling direction |
| $y_{w}, \dot{y}_{w}$ | Gradient perpendicular to the traveling direction and its time derivative |
| $a_{w}$, $b_{w}$ | Parameters to calculate the gradient perpendicular to the traveling direction |
| $q_{0}$ | Head-bending angle |
| *dc*(***x***_0_, *t*)/*dt* | Time derivative of the NaCl concentration sensed at the head tip of *C. elegans* |

Table S2 lists the parameters used in the multibody model.

**Table S2.** **Multibody model**

| Parameter | Value | Source |
| --- | --- | --- |
| Weight | 0.5 *μ*g | [14, 15] |
| Length | 0.10 mm |  |
| Radius | 40 *μ*m |  |
| Link number *L* | 12 | [23] |
| Normal friction^1)^ | 10 *μ*N mm/s | [4] |
| Tangential friction^1)^ | 1.5 *μ*N mm/s |  |

1. The friction parameters were set so that the body model travelled with an average speed of 1.2 mm/s

Table S3 shows the parameters used in the chemotaxis simulator derived from previous studies.

**Table S3.** **Parameters used to implement pirouette and weathervane**

| Parameter | Value | Source |
| --- | --- | --- |
| $q_{\mathrm{Max}}$ | 0.69 rad | [22] |
| $\omega$ | $2\pi\times0.80$ rad/s |  |
| $\psi$ | $1.54$ rad | [17] |
| $C_{w}$ | 1.374 rad/mM | [4] |
| $\Delta T$ | 12 s |  |
| $C_{r}$ | 0.35 rad |  |
| *T_b_* | 6.0 s | [21] |
| $T_{p1}$^1)^ | 1.0 s | [4] |
| $T_{p2}$^1)^ | 1.18 s |  |
| $T_{p3}$^1)^ | 1.0 s |  |

1. The total duration of a sharp turn is $T_{p1}+T_{p2}+T_{p3}=3.18$ s based on [2]

**S2 Appendix 2**

**A computational model of internal representations of chemical gradients in environments for chemotaxis of *Caenorhabditis elegans***

Zu Soh, Kazuma Sakamoto, Michiyo Suzuki, Yuichi Iino, Toshio Tsuji

**Fig S1. Definition of vectors and symbols related to head path and travelling direction**: The blue line indicates the head-bending path of the animal, the black line represents the travelling direction, ***x***_0_=(*x*_0_, *y*_0_) is the position of the head, ***v*** is the unit vector of the travelling direction, ***n*** is the unit orthogonal vector to the travelling direction, ***d*** is the head-bending velocity vector, and *θ* denotes the angle between vectors ***d*** and ***v***. The circle with grey gradation represents the NaCl concentration distribution, which is expressed by the function *c*(***x***, *t*). The two-dimensional coordinate of the agar surface is expressed as (*x*, *y*).

This appendix shows the derivation process of the proposed computational model (equations (1) and (2)). First, based on directional decomposition, we formulate the instantaneous relationship between the NaCl concentration sensed at the nose tip and the NaCl gradients parallel and perpendicular to the traveling direction. We then take the time average of this instantaneous relationship so that the formulation only includes the parameters accessible by the worm. Please note that we omit the explicit notation of time dependence of the variables for simplification, but all variables depend on time, unless stated otherwise. For example, the head-bending velocity vector depends on time, but we use ***d*** instead of ***d***(*t*). In the following equations, we use the dot operator ($\boldsymbol{\cdot}$) to express the inner product and the multiplication operator ($\boldsymbol{\times}$) to express the outer product.

**Step 1. Derive the instantaneous relationship between the NaCl concentration sensed at the nose tip and the NaCl gradients parallel and perpendicular to the traveling direction**

The sensory neurons ASEL/R located at the nose tip respond to the time derivative of NaCl concentration, which can be expressed by the following equation:

$$\frac{dc\left( \boldsymbol{x}_{0}\boldsymbol{,}t \right)}{dt}=\frac{\partial c\left( \boldsymbol{x}_{0}\boldsymbol{,}t \right)}{\partial\boldsymbol{x}_{0}}\frac{d\boldsymbol{x}_{0}}{dt}+\frac{\partial c\left( \boldsymbol{x}_{0}\boldsymbol{,}t \right)}{\partial t}$$

$$=\left[ \nabla_{x,y}c\left( \boldsymbol{x}_{0}\boldsymbol{,}t \right) \right]\boldsymbol{\cdot d+}\frac{\partial c\left( \boldsymbol{x}_{0}\boldsymbol{,}t \right)}{\partial t}, (s2-1)$$

where the symbols are defined in the figure legend of Fig. S1. The first term of equation (*s*2-1) indicates the directional derivative of the NaCl distribution toward the head-bending velocity vector ***d***, and the second term indicates the temporal change in the NaCl distribution. Here, the second term can be assumed to be much smaller than the first term because the animal moves much faster than the NaCl diffuses. Thus, we can focus on the first term and approximate the time derivative of NaCl concentration given to the ASEL/R neurons by the following equation:

$$\frac{\partial c\left( \boldsymbol{x}_{0}\boldsymbol{,}t \right)}{dt}\approx\left[ \nabla_{x,y}c\left( \boldsymbol{x}_{0}\boldsymbol{,}t \right) \right]\boldsymbol{\cdot d (}s2-2\boldsymbol{)}$$

If the travelling direction ***v*** and its orthogonal vector ***n*** are given, the time derivative of NaCl concentration at the nose tip (Equation ($s2-1)$)) can be decomposed into ***v*** and ***n*** direction components, which correspond to the gradients parallel and perpendicular to the travelling direction, respectively. This decomposition can be expressed by the following equations:

$$y_{p}\boldsymbol{\approx}\tilde{y}_{p}\boldsymbol{=}\left\{ \left[ \nabla_{x,y}c\left( \boldsymbol{x}_{0}\boldsymbol{,}t \right) \right]\boldsymbol{\cdot d} \right\}\boldsymbol{(d/|d|\cdot v}\mathbf{)}$$

$$\boldsymbol{=}\left\{ \left[ \nabla_{x,y}c\left( \boldsymbol{x}_{0}\boldsymbol{,}t \right) \right]\cdot\boldsymbol{d} \right\}\cos\theta. \left( s2-3 \right)$$

$$y_{w}\boldsymbol{\approx}\tilde{y}_{w}=\left\{ \left[ \nabla_{x,y}c\left( \boldsymbol{x}_{0}\boldsymbol{,}t \right) \right]\cdot\boldsymbol{d} \right\}\boldsymbol{(d/|d|}\cdot\boldsymbol{n)}$$

$$\boldsymbol{=}\left\{ \left[ \nabla_{x,y}c\left( \boldsymbol{x}_{0}\boldsymbol{,}t \right) \right]\cdot\boldsymbol{d} \right\}\boldsymbol{(d/|d|}\times\boldsymbol{v}\mathbf{)}$$

$$\boldsymbol{=}\left\{ \left[ \nabla_{x,y}c\left( \boldsymbol{x}_{0}\boldsymbol{,}t \right) \right]\cdot\boldsymbol{d} \right\}\sin\theta. (s2-4)$$

where $\tilde{y}_{p}$ and $\tilde{y}_{w}$ represent the approximated gradients parallel and perpendicular to the travelling direction, respectively. Equations (s2-3) and (s2-4) give the instantaneous NaCl gradients parallel and perpendicular to the traveling direction, respectively.

**2. Eliminate the directional parameter** $\theta$ **that is not accessible by the animal**

Equations (s2-3) and (s2-4) include the parameter $\theta$, which represents the angle between the head-bending direction $\boldsymbol{d/|d|}$ and the travelling direction $\boldsymbol{v}$, not likely accessible by the animal because the animal cannot observe the traveling direction on the global coordinates. Here, we take the time average of equations ($s2-3$) and ($s2-4$) (denoted by $\bar{y}_{p}$ and $\bar{y}_{w}$, respectively) to eliminate the parameter$\theta$:

$$\bar{y}_{p}=\frac{1}{t_{2}-t_{1}}\int_{t_{1}}^{t_{2}} \tilde{y}_{p}dt=\frac{1}{t_{2}-t_{1}}\int_{t_{1}}^{t_{2}} \left[ \nabla_{x,y}c\left( \boldsymbol{x}_{0}\boldsymbol{,}t \right) \right]\boldsymbol{\cdot d}\cos\theta dt$$

$$\bar{y}_{w}=\frac{1}{t_{2}-t_{1}}\int_{t_{1}}^{t_{2}} \tilde{y}_{w}dt=\frac{1}{t_{2}-t_{1}}\int_{t_{1}}^{t_{2}} \left[ \nabla_{x,y}c\left( \boldsymbol{x}_{0}\boldsymbol{,}t \right) \right]\boldsymbol{\cdot d}\sin\theta dt$$

Then, Steps 2.1 and 2.2 derive the NaCl gradient parallel and perpendicular to the travel direction, respectively.

**Step 2.1. The gradient parallel to the traveling direction**

Consider a time interval [*t*_1_, *t*_2_] where the sign of $\cos\theta$ does not change ($-\frac{\pi}{2}\leq\theta\leq\frac{\pi}{2}$), and $c_{\mathrm{low}}\leq\left[ \nabla_{x,y}c\left( \boldsymbol{x}_{0}\boldsymbol{,}t \right) \right]^{T}\boldsymbol{d\leq}c_{\mathrm{high}}$. The first mean value theorem for definite integrals indicates that there exist constants $c_{\mathrm{low}}\leq\tilde{\mu}_{p}\leq c_{\mathrm{high}}$ such that:

$$\bar{y}_{p}=\frac{1}{t_{2}-t_{1}}\int_{t_{1}}^{t_{2}} \left[ \nabla_{x,y}c\left( \boldsymbol{x}_{0}\boldsymbol{,}t \right) \right]\boldsymbol{\cdot d}\cos\theta dt=\tilde{\mu}_{p}\left\{ \frac{1}{t_{2}-t_{1}}\int_{t_{1}}^{t_{2}} \cos\theta dt \right\}. (s2-5)$$

Because $\theta$ changes periodically, $\frac{1}{t_{2}-t_{1}}\int_{t_{1}}^{t_{2}} \cos\theta dt$ becomes a constant value $B_{p}$ by setting the time interval [*t*_1_, *t*_2_] as the time duration of one $\theta$ cycle. Because $\theta$ is not accessible by the animal, we consider using the head-bending angle *q*_0_ instead. As $\theta$ approximately synchronizes with *q*_0_, *q*_0_ can be expressed by same frequency parameter *f* as $\theta$:

$$\theta=A_{\theta}\sin\left( 2\pi ft \right)$$

$$q_{0}=A_{q}\sin\left( 2\pi ft-\psi\right)$$

As the time interval [*t*_1_, *t*_2_] is the duration of one $\theta$ cycle, the following relationship holds:

$$B_{p}=\frac{1}{t_{2}-t_{1}}\int_{t_{1}}^{t_{2}} \cos\theta dt\approx\frac{A_{p}}{t_{2}-t_{1}}\int_{t_{1}}^{t_{2}} \cos q_{0}dt, (s2-6)$$

where *A_p_* is a gain constant. This time average operation thus allows us to eliminate $\theta$ from Equation (*s*2-5), and the equation can be rewritten as follows:

$$\bar{y}_{p}=\frac{1}{t_{2}-t_{1}}\int_{t_{1}}^{t_{2}} \left[ \nabla_{x,y}c\left( \boldsymbol{x}_{0}\boldsymbol{,}t \right) \right]\boldsymbol{\cdot d}\cos\theta dt=B_{p}\tilde{\mu}_{p}. (s2-7)$$

Here, we derive $\tilde{\mu}_{p}$ ($c_{\mathrm{low}}\leq\tilde{\mu}_{p}\leq c_{\mathrm{high}}$) by using the NaCl concentration sensed at the nose tip, approximated as $\left[ \nabla_{x,y}c\left( \boldsymbol{x}_{0}\boldsymbol{,}t \right) \right]\boldsymbol{\cdot d}$ in Equation (s2-2) by using the following leaky integration:

$$\frac{d\tilde{\mu}_{p}}{dt}=-a_{p}\tilde{\mu}_{p}+\tilde{b}_{p}\left[ \nabla_{x,y}c\left( \boldsymbol{x}_{0}\boldsymbol{,}t \right) \right]\boldsymbol{\cdot d}$$

where $a_{p}$ is the reciprocal of the time constant, and $\tilde{b}_{p}$ is the gain constant to scale the NaCl concentration sensed at the nose tip. These parameters depend on the time duration of integration *t*_2_- *t*_1_ and the scale of the NaCl concentration sensed at the nose tip. Substituting the above equation into the time differentiated (s2-7) gives

$$\frac{{d\bar{y}}_{p}}{dt}=B_{p}\frac{d\tilde{\mu}_{p}}{dt}$$

$$=-a_{p}B_{p}\tilde{\mu}_{p}+\tilde{b}_{p}B_{p}\left[ \nabla_{x,y}c\left( \boldsymbol{x}_{0}\boldsymbol{,}t \right) \right]\boldsymbol{\cdot d}$$

$$= -a_{p}\bar{y}_{p}+b_{p}\left[ \nabla_{x,y}c\left( \boldsymbol{x}_{0}\boldsymbol{,}t \right) \right]\boldsymbol{\cdot d}$$

where $b_{p}=B_{p}\tilde{b}_{p}$. The gradient parallel to the travelling direction can be obtained by solving this differential equation. Then, because we approximated the time derivative of the chemical concentration given to the sensory neurons by $\frac{dc\left( \boldsymbol{x}_{0}, t \right)}{dt}\approx b_{p}\left[ \nabla_{x,y}c\left( \boldsymbol{x}_{0}\boldsymbol{,}t \right) \right]^{T}\boldsymbol{d}$, the above equation can be rewritten to Equation (1):

$$\frac{dy_{p}}{dt}=-a_{p}y_{p}+b_{p}\frac{dc\left( \boldsymbol{x}_{0}, t \right)}{dt}+\epsilon_{p}, (1)$$

where the time constant ${1/a}_{p}$ smooths the time derivative of the NaCl concentration input $dc\left( \boldsymbol{x}_{0}, t \right)/dt$, and the gain constant $b_{p}$ scales the input. In addition, $\epsilon_{p}$ represents the accumulated error caused by the approximation errors in equation (*s*2-2), where the effect of NaCl diffusion is neglected, and equation (*s*2-6), where $\theta$ is replaced by $q_{0}$, and unexpected noises. This equation indicates that appropriate time averaging and scaling of the NaCl concentration sensed at the nose tip can generate the NaCl gradient parallel to the traveling direction.

**Step 2.2. The gradient parallel to the traveling direction**

A procedure similar to that described in step 2.1 can be used to derive equation (2) for calculating the gradient perpendicular to the travelling direction taking care of the sign of the sin function in Equation ($s2-4$) as follows:

$$\bar{y}_{w}=\frac{1}{t_{2}-t_{1}}\int_{t_{1}}^{t_{2}} \left[ \nabla_{x,y}c\left( \boldsymbol{x}_{0}\boldsymbol{,}t \right) \right]\boldsymbol{\cdot d}\sin\theta dt$$

$$=\tilde{\mu}_{w}^{+}\left\{ \frac{1}{\frac{t_{2}}{2}-t_{1}}\int_{t_{1}}^{\frac{t_{2}}{2}} \sin\theta dt \right\}+\tilde{\mu}_{w}^{-}\left\{ \frac{1}{t_{2}-\frac{t_{2}}{2}}\int_{\frac{t_{2}}{2}}^{t_{2}} \sin\theta dt \right\},$$

$$=B_{w}\left( \tilde{\mu}_{w}^{+}-\tilde{\mu}_{w}^{-} \right) (s2-8)$$

where $\theta>0$ in the time interval [$t_{1}, \frac{t_{2}}{2}]$, and $\theta\leq0$ in ($\frac{t_{2}}{2}, t_{2}]$. Using the head-bending angle *q*_0_ instead of $\theta$ gives

$$B_{w}=\frac{1}{\frac{t_{2}}{2}-t_{1}}\int_{t_{1}}^{\frac{t_{2}}{2}} \sin\theta dt=-\frac{A_{w}}{t_{2}-\frac{t_{2}}{2}}\int_{\frac{t_{2}}{2}}^{t_{2}} \sin q_{0}dt\approx\frac{A_{w}}{\frac{t_{2}}{2}-t_{1}}\int_{{t^{'}}_{1}}^{\frac{{t^{'}}_{2}}{2}} \sin q_{0}dt, (s2-9)$$

where $q_{0}>0$ in the time interval [${t'}_{1}, \frac{{t'}_{2}}{2}]$. The same holds for the time interval of [$\frac{t_{2}}{2}, t_{2}]$. Leaky integration for calculating $c_{\mathrm{low}}^{+}\leq\mu_{w}^{+}\leq c_{\mathrm{high}}^{+}$ and $c_{\mathrm{low}}^{-}\leq\mu_{w}^{-}\leq c_{\mathrm{high}}^{-}$ gives

$$\frac{{d\mu}_{w}^{+}}{dt}=-a_{w}\tilde{\mu}_{w}^{+}+\tilde{b}_{w}\left[ \nabla_{x,y}c\left( \boldsymbol{x}_{0}\boldsymbol{,}t \right) \right]\boldsymbol{\cdot d (}q_{0}>0)$$

$$\frac{{d\mu}_{w}^{-}}{dt}=-a_{w}\tilde{\mu}_{w}^{-}+\tilde{b}_{w}\left[ \nabla_{x,y}c\left( \boldsymbol{x}_{0}\boldsymbol{,}t \right) \right]\boldsymbol{\cdot d (}q_{0}\leq0)$$

Rewriting equation ($s2-4$) gives the following equations:

$$\frac{{d\bar{y}}_{w}}{dt}=B_{w}\left( \frac{d\tilde{\mu}_{w}^{+}}{dt}-\frac{d\tilde{\mu}_{w}^{-}}{dt} \right)$$

$$=\left\{ \begin{aligned} -B_{w}a_{w}\left( \tilde{\mu}_{w}^{+}-\tilde{\mu}_{w}^{-} \right)+B_{w}\tilde{b}_{w}\left[ \nabla_{x,y}c\left( \boldsymbol{x}_{0}\boldsymbol{,}t \right) \right]\boldsymbol{\cdot d} \left( q_{0}>0 \right) \\ -B_{w}a_{w}\left( \tilde{\mu}_{w}^{+}-\tilde{\mu}_{w}^{-} \right)-B_{w}\tilde{b}_{w}\left[ \nabla_{x,y}c\left( \boldsymbol{x}_{0}\boldsymbol{,}t \right) \right]\boldsymbol{\cdot d}\left( q_{0}\leq0 \right) \end{aligned} \right.$$

$$=\left\{ \begin{aligned} -a_{w}\bar{y}_{w}+b_{w}\left[ \nabla_{x,y}c\left( \boldsymbol{x}_{0}\boldsymbol{,}t \right) \right]\boldsymbol{\cdot d} \left( q_{0}>0 \right) \\ -a_{w}\bar{y}_{w}-b_{w}\left[ \nabla_{x,y}c\left( \boldsymbol{x}_{0}\boldsymbol{,}t \right) \right]\boldsymbol{\cdot d}\left( q_{0}\leq0 \right) \end{aligned} \right.$$

where $b_{w}=B_{w}\tilde{b}_{w}$. Because $\frac{dc\left( \boldsymbol{x}_{0}, t \right)}{dt}\approx b_{p}\left[ \nabla_{x,y}c\left( \boldsymbol{x}_{0}\boldsymbol{,}t \right) \right]^{T}\boldsymbol{d}$, the above equation can be rewritten as the proposed equation (2)

$$\frac{dy_{w}}{dt}=\left\{ \begin{matrix} -a_{w}y_{w}+b_{w}\frac{dc\left( \boldsymbol{x}_{0}, t \right)}{dt}\boldsymbol{+}\epsilon_{w}\boldsymbol{(}q_{0}>0) \\ -a_{w}y_{w}-b_{w}\frac{dc\left( \boldsymbol{x}_{0}, t \right)}{dt}\boldsymbol{+}\epsilon_{w}\boldsymbol{(}q_{0}\leq0) \end{matrix} \right. (2)$$

where ${1/a}_{w}$ is the time constant that smooths the time derivative of NaCl concentration input $dc\left( \boldsymbol{x}_{0}, t \right)/dt$, and $b_{w}$ is the gain constant to scale the input. Further, $\epsilon_{w}$ represents the accumulated error caused by the approximations in equations (*s*2-2) and (*s*2-9), and unexpected noises. This equation indicates that appropriate time averaging and scaling of the NaCl concentration sensed at the nose tip depending on the head-bending angle can generate the NaCl gradient perpendicular to the traveling direction.

# **S3 Supplemental Information**

**A computational model of internal representations of chemical gradients in environments for chemotaxis of *Caenorhabditis elegans***

Zu Soh, Kazuma Sakamoto, Michiyo Suzuki, Yuichi Iino & Toshio Tsuji

Figure S2 shows the pirouette of the body model and the animal. In this case, the pirouette performed by the model resulted in a change in the traveling direction by approximately 162°. The figure confirms that the body model can perform pirouette in the same manner as the animal. The turning angle largely depends on the posture when the sharp turn is initiated as well as the angle between the traveling direction and the NaCl peaks, as shown in Fig. S3.


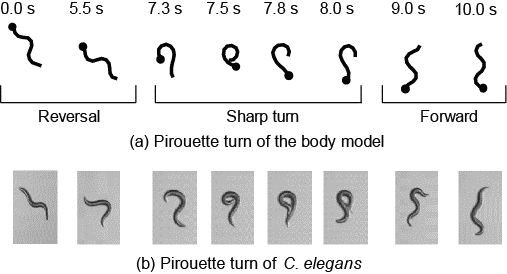


**Figure S2. Body posture of *C. elegans* during a pirouette.**

**(a)** Diagram of an example of pirouette performed by the multibody model of *C. elegans*. The reversal lasts approximately 6.0 s followed by a sharp turn lasting approximately 3.18 s, then the body model moves forward. These time parameters were derived from previous studies^2, 15^. **(b)** shows the pirouette turn performed by *C. elegans* to facilitate comparison between the movement of the multibody model and the actual worm.

Figure S3 compares the performance of weathervane and pirouette between the model and actual animal. Figure S3a shows the relationship between the spatial gradient and the average curving rate and Fig. S3b shows the distribution of bearing before the pirouette and the turning angles made by the sharp turns. Figure S3a confirmed that the weathervane index defined as the slope of the regression line is approximately 10.9, whereas that of the animal is approximately 12.0 according to the experimental data^2^. Figure S3b shows that both the simulation and animal make sharp turns with turning angles of approximately ±180° and the pirouette mostly occurs when the bearing angles are approximately ±180°.


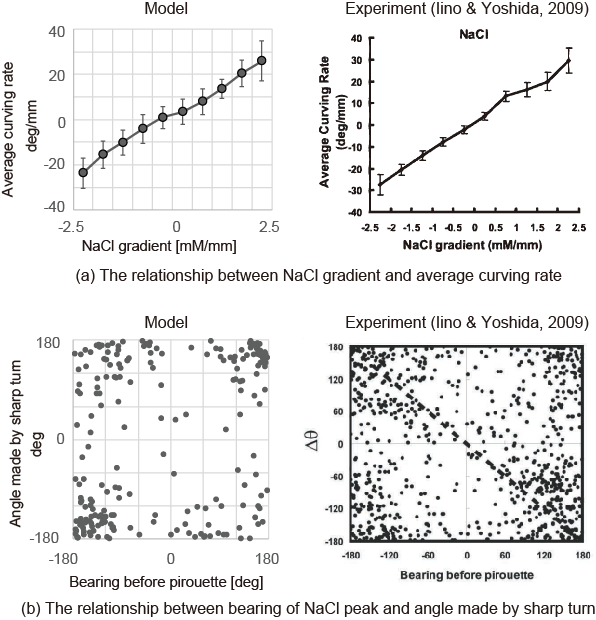


**Figure S3 Curving rate and bearing obtained from chemotaxis experiments^5^ and multibody model**

Graphs on the left show the simulation results and those on the right show the experimental data derived from the literature^2^.

**(a)** Weathervane performance of the model. The *x*-axis represents the spatial gradient and the y-axis denotes the average curving rate. The error bars show the standard deviation over results of 10 simulations. On the basis of the previous study^2^, the slope of the regression line was defined as the weathervane index, which was 10.9 for the simulation results and 12.0 for the animal.

**(b)** Pirouette performance of the model. The *x*-axis shows the bearing, which is defined as the angle between the traveling direction and the NaCl peak^2^, and y-axis represents the angles between the traveling directions before and after the sharp turn.

Next, to evaluate the movement generation error caused by the multibody model, we carried out a simulation driving the multibody model by using the measured postures of the actual animal as shown in the following steps:

(1) Video-recording of the wild-type animal

The wild-type (N2) of *Caenorhabditis elegans* was maintained on the 6-cm petri plate (IWAKI 60 mm/non-treated dish; AGC Techno Glass Co., Ltd., Shizuoka, Japan) containing 10 ml of nematode growth medium (NGM) spread with overnight-incubated *Escherichia coli* OP50 (food) as previously described^S1^. An adult *C. elegans* (3.5 days) was picked up from the culture plate and washed twice with S basal buffer solution^S1^. The washed animal was placed on a 3.5-cm petri plate (IWAKI 35 mm/non-treated dish) containing 3 ml of fresh NGM without food, and the locomotion was video-recorded for 10 s or more with a digital camera video-recorder (EX-F1, Casio Computer Co., Ltd, Tokyo, Japan) mounted on a stereomicroscope (SZX16, Olympus Corporation, Tokyo, Japan) with a frame rate of 300 fps and frame size of 640 x 480 pixels.

(2) Extracting the posture of the animal

By using the video analysis software specialized for *C. elegans* (Wriggle tracker, Library Inc., Tokyo, Japan) the centroid line of the body was extracted, and fit to the multibody model with 12 links of equal length. Then, the angles between adjacent links were extracted from each video frame. Figure S4 shows the extracted angles.

**Fig. S4. Extracted angles between adjacent links.** The vertical axis is time, the horizontal axis is the joint number, and the colour represents the angle corresponding to the colour bar shown on the right side. The animal showed reversal movement from around 2.5 s to 4 s, and performed an omega turn from around 4 s to 6 s.

(3) Driving the multibody model by using the extracted postures

The angles between adjacent links were substituted into the multibody model. The inverse dynamics problem was then solved to generate the traveling direction and the movement path. Figure S5 compares the paths of the multibody model and the animal.

**Figure S5. Comparison between the paths generated by the multibody model and the animal**. The green lines with circles show the posture of the multibody model where the circles represent the joints between the adjacent links. The blue line represents the head path of the worm, and the red line represents the head path of the multibody model.

We have also attached the video file of this analysis (MultiBodyModel.avi) where the posture of the multibody model is plotted with green lines over the recorded video of the animal.

(4) Evaluating the error

Finally, we evaluated the errors between the paths. Figure S6 shows the error at each frame between the head position extracted from the video and that obtained from the multibody model. Here, the position error at each frame is normalized by the body length of the animal (approximately 1.03 mm).

**Fig. S6. Position error between the head position extracted from the video and that obtained from the multibody model.** The result shows that the position error is less than 1% of body length.

This simulation result suggests that the head path error caused by the multibody model is sufficiently small.

**Reference**

**S1.** Brenner S. The genetics of *Caenorhabditis elegans*. *Genetics* **77,** 71–94 (1974).
